# 

**Published:** 1895-09-07

**Authors:** 


					^ADBURY'S ^ WW CADBURY'S
0tab?iM, COCOA rip J^i COCOA
P?"ty and freedom from alkali." Jg^ <*?!> <2? jMO* " The Typical Cocoa of English Manufacture^^ *
?Braithwaite. ' Absolutely Pure."? The Analyst* / \\
UscotT , ?<3^ ? -?mnLKA^ NORWICH HOSPITAL
5?- 4?- Vol. xvm. WITH EXTRA NURSING SUPPLEMENT. pP3SSL.3KS^?E-
*t>AY, Stttt 7ttt 1 RCK [Registered at the General Post Offioe as a Newspaper for Monthly Parts, 6d.; post free, 8d.
? ' A* ' 1 ll> low' tranamission in the United Kingdom andAbroad.] Ditto per Annnm, 8s.Ud., poat free.

				

